# Supplementary material for: Clinical Predictors for Procedural Stroke and Implications for Embolic Protection Devices during TAVR: Results from the Multicenter Transcatheter Aortic Valve Replacement In-Hospital Stroke (TASK) Study
Source: J Pers Med. 2022 Jun 28;12(7):1056. doi: 10.3390/jpm12071056 (PMC9316224; doi:10.3390/jpm12071056)
Supplement: Supplementary file 1 [file jpm-12-01056-s001.zip › jpm-1745451-supplementary.pdf]

Supplementary

Table S1. Participating centers and personnel.

Center	Personnel
Division of Cardiology, Leviev Heart and Vascular Center, Chaim Sheba Medical Center, Tel Hashomer, Israel	Anat Berkovitch<br>Elad Maor<br>Israel M. Barbash<br>Amit Segev
Rabin Medical Center, Petach-Tikva, Israel	Katia Orvin<br>Ran Kornowski
Division of Cardiology, Tel Aviv Medical Center, Tel Aviv, Israel	Ariel Finkelstein<br>Arie Steinvil
Department of Interventional Cardiology, Clinique Pasteur, Toulouse, France	Didier Tchétché<br>Pierre Robert
Heart Center Bonn, Germany	Alexander Sedaghat<br>Jan-Malte Sinning<br>Georg Nickenig
Cardiovascular Surgery Department, University Hospital of Zurich, Zurich, Switzerland	Matteo Saccocci<br>Francesco Maisano
Interventional Cardiology Unit, San Raffaele Scientific Institute, Milan, Italy	Azeem Latib<br>Gianmarco Iannopollo
Interventional Cardiology Department, Hospital Universitario Marques de Valdecilla, Santander, Spain	Jose M De la Torre Hernandez<br>Dae Hyun-Lee
The Heart Center, Rigshospitalet, Blegdamsvej 9, 2100 Copenhagen, Denmark	Lars, Søndergaard<br>Ole De Backer
University Hospital and SAOLTA Health Care Group, National University of Ireland, Galway, Ireland	Darren Mylotte<br>Federico Mercanti
Department of Cardiology, Radboud University Medical Center, Postbus 9101, 6500 HB Nijmegen, The Netherlands	Niels van Royen<br>Kees van der Wulp
Cardiology, Freeman Hospital and Institute of Cellular Medicine, Newcastle University, United Kingdom	Azfar G Zaman<br>Joy Shome

Table S2. Multivariate logistic regression analysis for the risk of peri-procedural cerebrovascular events.

**Original Data**			
	**Odds Ratio**	**Confidence Interval**	***p*-Value**
History of previous stroke	1.84	1.01–3.34	0.046
Non-balloon expandable valves	2.06	1.29–3.30	0.002
Peripheral artery disease	1.82	1.19–2.80	0.006
Chronic kidney disease	2.14	1.31–3.51	0.002
Bootstrap data			
History of previous stroke	1.92	1.01–3.19	
Non-balloon expandable valves	2.134	1.32–3.50	
Peripheral artery disease	1.81	1.18–2.68	
Chronic kidney disease	2.26	1.37–4.03	

Table S3. C statistic, optimism-corrected C statistic, and cross-validated C statistic.

.	C Statistic
Original data	0.658
Cross-validation	0.641
Optimism of C statistics	0.00838

Table S4. Multivariate Cox regression analysis for the risk of mortality at 1 year.

Variable	Hazard Ratio	Confidence Interval	*p*-Value
Age	1.00	0.98–1.01	0.49
Female gender	1.04	0.88–1.24	0.62
Ischemic heart disease	1.89	1.53–2.34	<0.001
Diabetes mellitus	1.30	1.09–1.55	0.003
Hypertension	1.01	0.83–1.23	0.94
Peripheral vascular disease	1.42	1.16–1.74	0.001
Chronic kidney disease	1.52	1.25–1.85	<0.001
Ejection fraction	0.99	0.98–0.99	0.013
Previous stroke	1.81	1.25–2.62	0.002
Peri-procedural cerebrovascular event	1.78	1.06–2.98	0.028
